# Hæmatoma in a Cow

**Published:** 1890-01

**Authors:** W. H. Ridge

**Affiliations:** Trevose, Penna.


### HÆMATOMA IN A COW.

By W. H. Ridge, V.M.D.,
Trevose, Penna.

Nov. 11th, I saw a cow belonging to a farmer. There was a tumor on the inside of off front leg, extending from humero-radial articulation downward, and is the size of a cocoanut, hard, tense to feel, gives pain when manipulated, no point of fluctuation, leg a little swollen below, cannot get pulsation, or feel the pulse below the tumor: cow is with calf, and in fair condition. I punctured with small trocar canula, ⅛ in., when I got a profuse hemorrhage, and thought perhaps I might have entered the post-radial; I punctured again in another place, with the same result, but on pressure, gave such pain that I discontinued its use.

Nov. 24, I saw the case again. Tumor twice as large as before, and the animal suffering. I explored again, and found that by entering the trocar about one inch I got no hemorrhage, but could push the canula into it as far as desired without the use of stillett. When pushing in farther—about 2½ inches—I got the same hemorrhage.

Dec. 6. Tumor extends from elbow to carpus, about one foot in thickness; cow is emaciated, cannot use the affected leg; I explored again; at first got black coagulated blood, on pushing canula further, and moving the end about, I got hemorrhage again; ordered her killed.

*Autopsy.*—Skin attached to tumor, tumor fast to radius and muscles, so that I had to take some of the muscles with tumor; found the radial arteries enlarged, but they did not enter the tumor directly on dissection of both arteries from tumor. I found several branches running into it; on opening tumor, a mass of black, coagulated blood filled the whole cavity, except in a spot in the center, which had hard coagulated blood. The clot was laminated, with very firm walls, and seemed made up of channels on the inside, while the outside layer was composed of dense, fibrous tissue. The humeral lymphatic glands were enlarged; lungs, liver, kidneys. and other parts healthy.

